# Sex Specific Changes in Tryptophan Breakdown Over a 6 Week Treatment Period

**DOI:** 10.3389/fpsyt.2019.00074

**Published:** 2019-02-21

**Authors:** Eva Z. Reininghaus, Nina Dalkner, Karin Riedrich, Dietmar Fuchs, Johanna M. Gostner, Bernd Reininghaus

**Affiliations:** ^1^Department of Psychiatry and Psychotherapeutic Medicine, Medical University of Graz, Graz, Austria; ^2^TZ-Justus Park Bad Hall, Bad Hall, Austria; ^3^Division of Medical Biochemistry, Biocenter, Medical University of Innsbruck, Innsbruck, Austria; ^4^Division of Biological Chemistry, Biocenter, Medical University of Innsbruck, Innsbruck, Austria

**Keywords:** tryptophan, kynurenine, sex, sex differences, body mass index

## Abstract

**Introduction:** Despite the knowledge of sex differences concerning neurobiological parameters as well as clinical course of illness in individuals with mood disorders, the literature concerning tryptophan (Trp) breakdown, specific for women and men, is sparse to date. The current study aimed to evaluate sex differences in Trp, kynurenine (Kyn) and Kyn/Trp concentrations in general, as well as differences in changes of those concentrations over the course of a 6-week rehabilitation program in individuals with life-time unipolar affective disorder. For this purpose changes in Trp and Kyn as well as the Kyn/Trp concentrations between the time of admission (t1) and discharge (t2) were analyzed in dependence of sex. Furthermore, correlations between Trp and Kyn levels and clinical parameters were performed separately for male and female participants.

**Material and Methods**:

**Results:** For the current analysis 426 individuals with lifetime affective disorder completing a 6-week rehabilitation program were included. In both sexes, psychiatric symptoms decreased significantly over time. There was a significant difference between women (*n* = 242) and men (*n* = 184) regarding the changes in Trp, Kyn, and Kyn/Trp over time even if controlled for relevant covariates [multivariate: *F*_(3, 380)_ = 2.663, η^2^ = 0.021, *p* = 0.048]. Kyn as well as Kyn/Trp concentrations increased significantly in men over time (Kyn *F* = 4.809, η^2^ = 0.012, *p* = 0.029; Kyn/Trp *F* = 7.923, η^2^ = 0.020, *p* = 0.005). Results remained the same when controlled for psychiatric symptoms.

**Discussion:** The main finding of the present study is the significant difference between women and men regarding the change in Trp, Kyn, and Kyn/Trp over a 6-week psychiatric treatment period, while the depression severity scores as well as general psychiatric symptoms decreased. Sex specific changes in Trp-Kyn pathways have only been explored to a very small extent to date in the literature but are of high clinical relevance in the context of personalized medicine.

## Introduction

In recent decades, the association between the tryptophan (Trp)-kynurenine (Kyn) axis, low-grade inflammation and neuropsychiatric disorders has been shown in various studies. The Kyn/Trp ratio reflects levels of Trp breakdown and can be used as a proxy measure of indoleamine 2,3-dioxygenase-1 (IDO) activity. An increase in IDO activity is usually driven by chronic low-inflammatory processes which breaks Trp down the Kyn pathway and reduces serotonin and melatonin synthesis (1). A number of studies have proven the role of IDO in cytokine-induced depression by demonstrating correlations between cytokine-induced depression and lower Trp and raised Kyn concentrations as well as an increase in the Kyn/Trp-ratio (2, 3). Originally, the role of such processes has been shown in depressive symptoms accompanying interferon-treated hepatitis C patients (3–6).

### Sex Differences

Sex differences in concentrations of clinical and neurobiological biomarkers have been reported in a multitude of studies concerning affective disorders (7–12); however, data on sex specific differences concerning the Trp-Kyn axis and psychiatric disorder are sparse to date. We could recently show a shift toward the (neurotoxic) hydroxykynurenine arm of the kynurenine pathway in adult euthymic males with bipolar disorder and an association with poorer performance in short- and long-term verbal memory (9). Those associations were not present in female individuals with bipolar disorder and point toward the involvement of the Trp-Kyn axis in sex-specific cognitive dysfunction of psychiatric diseases.

Despite the high number of studies investigating the Trp-Kyn pathway in individuals with neuropsychiatric symptoms undergoing cytokine treatment, studies in psychiatric cohorts are comparatively rare and results contradictory. As an example, a recent publication (13), as well as some older studies (14–16) found increased Kyn/Trp in depressive individuals compared to controls. In contrast, other studies found decreased Kyn and a decreased Kyn/Trp in depressed individuals compared to healthy controls (17, 18). Nevertheless, in a cohort study, no correlations between depressive symptoms and Kyn/Trp could be found (17). Inflammatory parameters correlated in most of the studies with Kyn/Trp, but inflammation did not mediate the association between Kyn/Trp and depressive symptoms (17).

Measuring clinical and neurobiological changes over time during a therapeutic process requires a very structured treatment with a predefined treatment plan that applies the same therapy levels to all patients. Psychiatric rehabilitation settings provide an intensive, multidisciplinary 6-week program for individuals with serious mental illness, most typically suffering from affective disorders and due to the structured program, treatment outcome can be easily analyzed. In this setting, the current symptoms of patients are not serious enough to require acute psychiatric care; most patients receive rehabilitative treatment following acute therapy in an psychiatric hospital. Current acute and severe psychopathology is not necessarily prevalent in all patients. The rehabilitation program is a structured and targeted setting which includes medical, psychiatric, psychological and psychotherapeutic treatments, as well as occupational therapy, physiotherapy and diet counseling. The principal goals of the rehabilitation setting include long-term management of psychiatric symptom, strengthening of social skills, active participation in everyday life, improving of cognitive functioning, and decreasing the rates of re-hospitalization and retirement due to disability (19). We recently showed that there is an association between the therapeutic response to this multimodal treatment and changes in Kyn concentrations, the Kyn/Trp ratio as well as high sensitive C-reactive protein (hsCRP) in severely depressed patients not receiving cytokine treatment (20). Importantly, Kyn increased significantly in the patient group not responding to treatment, while the Kyn/Trp ratio decreased significantly in the group of responders over time. In addition, changes in Kyn as well as hsCRP concentrations correlated significantly with changes in the body mass index (BMI) over time in this study. Nevertheless, sex specific aspects could not be considered due to small subgroup sample sizes.

Despite the knowledge of sex differences concerning neurobiological biomarkers as well as clinical course of illness in individuals with mood disorders, the literature concerning Trp breakdown, specific for women and men, is sparse to date. The current study aimed to evaluate sex differences in Trp, Kyn, and Kyn/Trp concentrations in general, as well as differences in changes of those concentrations over the course of a 6-week rehabilitation program in individuals with life-time unipolar affective disorder in an exploratory study design. As a unidirectional differential hypothesis we formulated that there is a significant difference in the change of Trp, Kyn, and Kyn/Trp concentrations over a 6-week rehabilitation treatment setting between women and men. For this purpose, changes in Trp and Kyn concentrations as well as the Kyn/Trp ratio between the time of admission (t1) and discharge (t2) were analyzed in dependence of sex. Furthermore, correlations between Trp and Kyn concentrations and clinical parameters were performed separately for male and female participants.

## Materials and Methods

### Participants

The study was conducted at a psychiatric rehabilitation center with treatment focus on affective and stress-related disorders. Data of 600 individuals treated between April 2015 and April 2017 were available, of whom 426 patients had a life-time diagnosis of unipolar affective disorder (F32 and F33 according to ICD-10 diagnosis) and were included in the current analysis. The treating psychiatrist performed the respective diagnoses according to the ICD-10 diagnosis criteria [International classification of mental disorders (21)] using record reviews. None of the patients had a substance use disorder as a main diagnosis, as this was an exclusion criteria for taking part in the rehabilitation setting in general. All participants completed the 6-week rehabilitation program, consisting of weekly medical consultations, psychotherapy (single and group setting), occupational therapy, physiotherapy, physical training as well as diet counseling. Every patient had to attend 18–20 h of therapy per week, this was performed either in a single or a group setting. In general all patients attending psychiatric rehabilitation received a very similar structured and targeted program. The patients undergoing the psychiatric rehabilitation program differed in their stage of recovery. In general, the psychiatric rehabilitation setting follows acute psychiatric inpatient treatment protocols; however waiting times for therapy program initiation are different and dependent on insurance approval. The whole study enrolled current psychiatric symptoms, complete lifetime psychiatric history, anthropometric measure, fasting blood, psychological testing and various lifestyle questionnaires, data were collected by trained clinic staff.

The study has been approved by the local ethics committee of the Medical University of Linz, Austria, in accordance with *The Code of Ethics* of the World Medical Association (Declaration of Helsinki 1964; General Assembly of the World Medical Association, 2014), ICH guideline for Good Clinical Practice and current regulations (EK-number: E-24-14). Written consent was obtained from all participants at the time of admission and all received the same study procedure.

### Psychological Inventories

Depressive symptoms were assessed with the Hamilton Depression Scale (HAM-D) (22) and the Beck Depression Inventory (BDI-II) (23). The Symptom-Checklist Revised (SCL-90-R; (24), a 90-item self-report inventory, was used to assess a broad range of psychological symptoms and psychological distress in the last 7 days. Cognitive, physical, and emotional symptoms of distress, and overall distress were rated on a five-point Likert scale with 53 items and the Global Severity Index (GSI) was used for the current analysis to capture fundamental psychological distress.

### Biological Assays

For the measurement of serum inflammatory markers and amino acids, fasting blood samples were collected between 8.00 and 10 a.m., samples were either processed immediately for further analyses (hsCRP, IL-6) or stored at −80°C until thawed for the biological assays. Free Trp and Kyn serum concentrations were determined by high-performance liquid chromatography, as described earlier (25). Kyn/Trp was calculated as index of Trp breakdown providing a proxy of IDO activity (if it correlates with markers of immune activation). Levels of hsCRP were analyzed by Fa Abbott with Architect ci8200 and IL-6 was analyzed by Fa Roche with Cobas e411.

### Statistics

#### Description of the Cohort at the Time of Admission

Due to distribution of data, nonparametric tests were used. For comparing means at t1, Mann-Whitney-U-Tests were used. Additionally, Spearmann-correlations between Trp, Kyn, and Kyn/ Trp and clinical variables at t1 were calculated. A multivariate analysis of co-variance (MANCOVA) with sex as the between subject factor and the concentration of Trp, Kyn, and Kyn/Trp at t1 as dependent variables was performed. Age, BMI, interleukin-6 (IL-6), GSI and cardiovascular disease were inserted as covariates as they differed between women and men at the time of admission. Moderation analysis was used to calculate if sex significantly moderates the correlations between depressive symptoms measured with BDI-II as well as HAM-D and metabolites at the time of admission (moderator variable = sex, independent variable = BDI-II or HAM-D, dependent variable = respective metabolite).

Concerning medical comorbidities, cardiovascular disease (including hypertension) was significantly more often found in men (*p* = 0.004, 53% vs. 38%), while there was no difference in the prevalence of diabetes mellitus or lipid associated diseases between men and women in our cohort.

#### Changes Over Time

Changes of the respective variables between t1 and t2 were calculated as a mean difference (respective variable_Diff). For statistical analyses we conducted a multivariate analysis of co-variance (MANCOVA) with sex as the between subjects factor and the changes of Trp, Kyn and Kyn/Trp as dependent variables. Age, BMI, IL-6 and cardiovascular disease were inserted as covariates as they differed between women and men at the time of admission (see Table 1). In a further step, psychiatric symptoms, namely HAM-D_Diff as well as GSI and BDI, were introduced as covariates. Within the sex groups, non-parametric Wilcoxon Rank Sum Test was used to analyze changes in Trp, Kyn, and Kyn/ Trp over time. Non-parametric correlations (Spearmann-Rho) between the mean change of Trp, Kyn, and Kyn/Trp between t1 and t2 and the changes in clinical variables between t1 and t2 were analyzed. Moderation analysis was used to test whether sex can significantly moderate a possible correlation between depressive symptoms measured with BDI as well as HAM-D and differences in metabolites over time. Error probabilities below 0.05 were accepted to denote statistical significance for *t*-tests and multivariate analyses. Additionally, due to the multitude of clinical variables, variables at *p* < 0.004 are given for correlation analyses to mark Bonferroni corrected values.

**Table 1:** *Descriptive statistics* of women and men at the time of admission.

**T1**	**Females *M* (*SD*)**	**Males*M* (*SD*)**	**Differences between women and men**
Age [years]	53.6 (7.8)	51.7 (8.0)	*z* = −3.0, *p* = 0.003^**^
BMI [kg/m2]	26.0 (5.0)	27.2 (4.1)	*z* = −3.0, *p* = 0.002^**^
Smoking severity	0.81 (1.8)	1.1 (2.1)	n.s.
Cardiovascular disease (%)	38.2%	52.5%	*t* = −2.9, *p* = 0.004^**^
hsCRP	2.1 (2.1)	2.0 (2.0)	n.s.
IL-6	2.1 (2.1)	2.8 (3.0)	*z* = −2.5, *p* = 0.012^*^
BDI-II	21.8 (9.8)	20.0 (10.8)	n.s.
HAM-D	11.3 (6.0)	12.4 (7.4)	n.s.
GSI	1.1 (0.6)	1.0 (0.6)	*z* = −2.0 *p* = 0.043^*^
Trp t1 [μmol]	62.4 (7.6)	68.0 (8.0)	*F*_(3, 407)_ = 15.154, *p* = 0.000^**^ Trp *F* = 43.0, *p* = 0.000^**^
Kyn t1 [μmol]	1.8 (0.47)	1.9 (0.42)	Kyn *F* = 1.4, *p* = 0.241
Kyn/Trp t1 [μmol]	0.0298 (0.0074)	0.0281 (0.0057)	Kyn/Trp *F* = 6.5, *p* = 0.011^*^

* significant for p < 0.05,

** significant at p < 0.01; BMI, body mass index; hsCRP, high sensitive reactive protein; IL-6, interleukin 6; BDI-II, Becks Depression Inventory; HAM-D, Hamilton Depression Score; GSI, Global severity index (SCL); Trp, Tryptophan; Kyn, Kynurenine.

## Results

### Time of Admission

Participants had a mean age of 52.8 (SD 8.0) and a mean BMI of 26.5 (SD 4.7), 56.8% (*n* = 242) were female. Of all participants, 29.3% had university or polytechnic degree, 19.4% were divorced and 13.4% were single without a partner. 33.3% did exercise at least once a week, 43.4% had a therapy with Serotonin reuptake inhibitors, 23.2% with serotonin and norepinephrine reuptake inhibitors and 12.2% with atypical antipsychotics. Men had significantly lower age and higher levels in the GSI, higher BMI, IL-6 and more cardiovascular disease as well as higher Trp and lower Kyn/Trp compared to females. There was no difference in smoking severity (Fagerstroem Test for nicotine dependence), depressive symptoms or hsCRP levels at the time of admission. Further descriptive data for both groups at the time of admission are displayed in Table 1.

In both sexes, there was a significant correlation between Kyn and age as well as BMI. Furthermore, there was a significant correlation between Kyn/Trp and age as well as hsCRP. In women only, we found a significant correlation between Kyn and hsCRP as well as between Kyn/Trp and IL-6. In men only, we found a significant correlation between Kyn/Trp and BMI as well as a negative correlation between Kyn and BDI-II. Moderation analyses showed that sex did not significantly moderate the correlation between depressive symptoms and Trp, Kyn as well as Kyn/Trp at the time of admission.

Table 2 gives the associations between Trp, Kyn, and Kyn/Trp concentrations and clinical parameters at t1. Table 3 gives an overview about the associations at t2.

**Table 2:** Associations of serum tryptophan and kynurenine concentrations with clinical parameters at the time of admission.

	**Women**			**Men**		
	**Trp [μmol/L]**	**Kyn [μmol/L]**	**Kyn/Trp [μmol]**	**Trp [μmol/L]**	**Kyn [μmol/L]**	**Kyn/Trp [μmol]**
Age [years]	*r =* −0.025 *p* = 0.697	***r*** **=** 0.298^**^ p **=** **0.000**	***r*** **=** 0.308^**^ p **=** **0.000**	*r* = −0.131 *p* = 0.077	***r*** **=** 0.241^**^ p **=** **0.001**	***r*** **=** 0.331*** p **=** **0.000**
BMI [kg/m2]	*r* = 0.151^*^ p = 0.020	***r*** **=** 0.238^**^ p **=** **0.000**	*r* = 0.161^*^ p = 0.013	*r* = 0.047 *p* = 0.526	***r*** **=** 0.300*** p **=** **0.000**	***r*** **=** 0.269*** p **=** **0.000**
Smoking severity	*r* = 0.078 *p* = 0.228	*r* = −0.79 *p* = 0.222	*r* = −0.128 *p* = 0.048^*^	*r* = −0.009 *p* = 0.909	*r* = −0.176 *p* = 0.019^*^	*r* = −0.195 *p* = 0.009^*^
BDI-II	*r* = −0.121 *p* = 0.064	*r* = −0.123 *p* = 0.059	*r* = −0.80 *p* = 0.221	*r* = −0.188^*^ p = 0.012	***r*** **=** –0.243^**^ p **=** **0.001**	*r* = −0.165^*^ p = 0.028
HAM-D	*r* = −0.143^*^ p = 0.035	*r* = −0.081 *p* = −231	*r* = 0.007 *p* = 0.920	*r* = −0.145 *p* = 0.058	*r* = −0.181^*^ p = 0.018	*r* = −0.113 *p* = 0.140
GSI	*r* = −0.159^*^ p = 0.014	*r* = −0.124 *p* = 0.056	*r* = −0.68 *p* = 0.298	*r* = −0.116 *p* = 0.119	*r* = −0.115 *p* = 0.123	*r* = −0.043 *p* = 0.567
hsCRP	*r* = 0.093 *p* = 0.148	***r*** **=** 0.216^**^ p **=** **0.001**	***r*** **=** 0.189^**^ p **=** **0.003**	*r* = −0.112 *p* = 0.128	*r* = 0.186^*^ p = 0.012	***r*** **=** **0.229** ***p*** **=** 0.002^**^
IL-6	*r* = −0.024 *p* = 0.707	*r* = 0.163^*^ p = 0.011	*r* = 0.183 *p* = 0.004^*^	*r* = −0.150 *p* = 0.042	*r* = 0.073 *p* = 0.322	*r* = 0.163^*^ p = 0.027

* significant at p < 0.005;

** significant if corrected for Bonferroni at p < 0.004; BMI, body mass index; hsCRP, high sensitive reactive protein; IL-6, interleukin 6; BDI-II, Becks Depression Inventory; HAM-D, Hamilton Depression Score; GSI, Global severity index (SCL); Trp, Tryptophan; Kyn, Kynurenine.

**Table 3:** Descriptive statistics of women and men at the time of discharge.

**T2**	**Women**	**Men**	
BMI t2 [kg/m2]	25.7, *SD* = 4.7	26.8, *SD* = 3.8	*t* = 2.8, *p* = 0.005**
hsCRP	1.8 (1.9)	1.7 (1.7)	n.s.
IL-6	2.3 (1.8)	2.7 (2.2)	*t* = 2.0, *p* = 0.042*
BDI-II t2	10.9 (10.4)	11.1 (10.1)	n.s.
HAM-D t2	7.4 (5.7)	7.2 (5.7)	n.s.
GSI	0.6 (0.5)	0.6 (0.5)	n.s.
TRP t2 [μmol]	62.7, *SD* = 8.5	68.3, *SD* = 8.5	*F*_(3, 395)_ = 12.6, *p* = 0.000** (corrected for age, BMI, IL-6) Trp *F* = 37.6, *p* = 0.000**
KYN t2 [μmol]	1.8 (0.46)	2.0 (0.42)	Kyn *F* = 10.9, *p* = 0.001**	
KYN/TRP t2 [μmol]	0.0292 (0.0068)	0.0290 (0.0056)	Kyn/Trp *F* = 0.094, *p* = 0.760	

* *significant for p < 0.05*,

** *significant at p < 0.01; BMI, body mass index; hsCRP, high sensitive reactive protein; IL-6, interleukin 6; BDI-II, Becks Depression Inventory; HAM-D, Hamilton Depression Score; GSI, Global severity index (SCL); Trp, Tryptophan; Kyn, Kynurenine*.

### Changes Over Time

In both sexes, psychiatric symptoms decreased significantly over time (HAM-D *M* = −4.641, *SD* = 0.510, BDI *M* = −10.519, *SD* = 9.538, GSI *M* = −1.141, *SD* = 0.510). Reductions of HAM-D scores were significantly higher in men (*M* = −5.262, *SD* = 5.076) compared to women over time (*M* = −4.133, *SD* = 4.584). BMI, BDI-II, GSI, and hsCRP levels decreased significantly over time in both sexes, but there were no significant differences in the changes over time between women and men. The levels of IL-6 remained stable over time in both sexes.

There was a significant difference between women and men regarding the change in Trp, Kyn, and Kyn/Trp over time even if controlled for age, BMI, IL-6 and cardiovascular disease [multivariate: *F*_(3, 380)_ = 2.663 *p* = 0.048, η^2^ = 0.021]. Kyn as well as Kyn/Trp increased in men compared to women (Kyn_Diff *F* = 4.809, *p* = 0.029, η^2^ = 0.012; Kyn/Trp_Diff *F* = 7.923, *p* = 0.005, η^2^ = 0.020). Wilcoxon Rank Sum Test showed that Kyn and Kyn/Trp increased significantly in men (Kyn_Diff *z* = −2.077, *p* = 0.038; Kyn/Trp_Diff *z* = −2.307, *p* = 0.21).When psychiatric symptoms were included in the multivariate analysis (GSI, BDI, HAM_Diff) the results remained the same [multivariat: *F*_(3, 346)_ = 2.796, *p* = 0.040, η^2^ = 0.024] with similar significant changes in Kyn_Diff as well as Kyn/Trp_Diff. Moderation analysis showed that the interaction of the change in objective depressive symptoms (HAM_Diff) and sex significantly moderated the change in Kyn/Trp over time [*F*_(3, 382)_ = 5.571, *p* = 0.001; interaction effect: β = 0.107, *T* = 2.099, *p* = 0.036].

**Figure 1:**
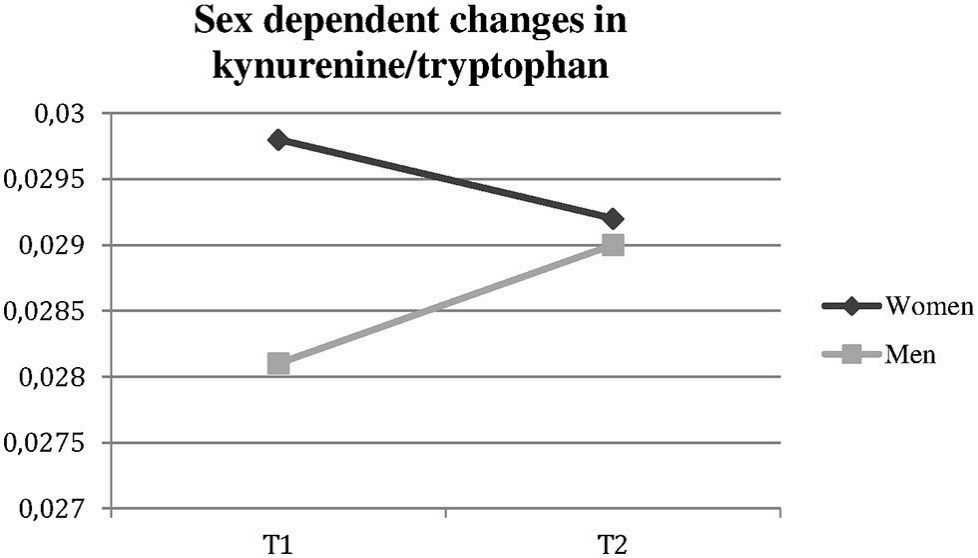
Significantly different changes in kynurenine/tryptophan ratio in women vs. men over time. t1, time of admission; t2, time of discharge.

**Figure 2:**
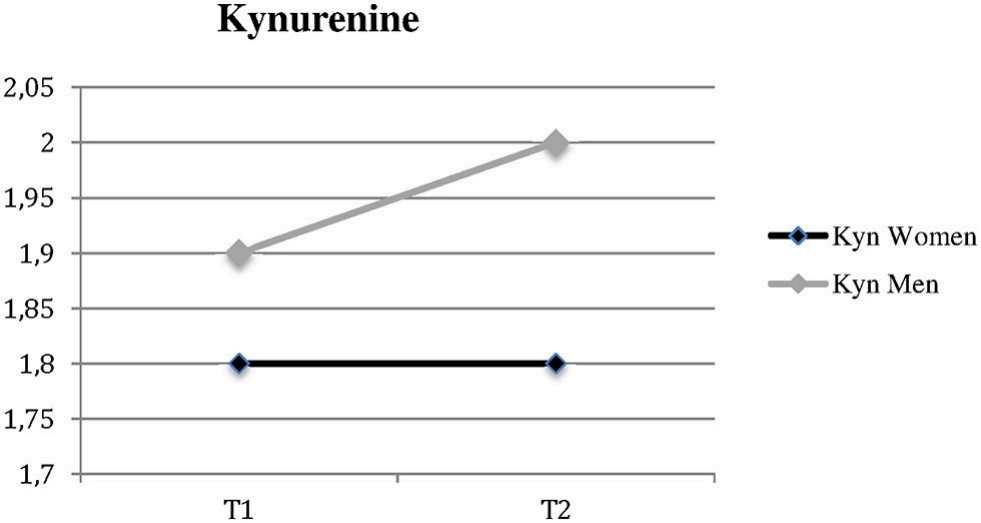
Significantly different changes in kynurenine in women vs. men over time Kyn, Kynurenine; t1, time of admission; t2, time of discharge.

The median Kyn and Kyn/Trp changes in men and women are shown in Figures 1, 2.

There were significant positive correlations between the changes in BMI over time and changes in Trp as well as Kyn concentrations in women and between BMI changes and changes in Kyn as well as Kyn/Trp in men (Bonferroni corrected) if only Bonferroni corrected values were included. Further results with lower significance can be obtained from Table 4.

**Table 4:** Associations between changes of serum tryptophan and kynurenine concentrations over time.

	**Women**			**Men**		
	**Trp _Diff**	**Kyn_Diff**	**Kyn/ Trp _Diff**	**Trp _Diff**	**Kyn _Diff**	**Kyn/ Trp_Diff**
BMI_Diff	***r*** **=** 0.286** p **=** **0.000**	***r*** **=** 0.288** p **=** **0.000**	*r* = 0.097 *p* = 0.147	*r* = 0.185* p = 0.014	***r*** **=** 0.357** p **=** **0.000**	***r*** **=** 0.244** p **=** **0.001**
Smoking severity	*r* = 0.007 *p* = 0.917	*r* = 0.072 *p* = 0.281	*r* = 0.099 *p* = 0.140	*r* = 0.195* p = 0.010	*r* = 0.163* p = 0.032	*r* = −0.019 *p* = 0.800
BDI_Diff	*r* = 0.077 *p* = .0291	*r* = 0.053 *p* = 0.472	*r* = 0.034 *p* = 0.642	*r* = −0.024 *p* = 0.772	*r* = 0.077 *p* = 0.355	*r* = 0.092 *p* = 0.267
HAM-D_Diff	*r* = −0.097 *p* = 0.160	*r* = −0.178* p = 0.009	*r* = −0.090 *p* = 0.189	*r* = −0.076 *p* = 0.323	*r* = –0.024 *p* = 0.752	*r* = 0.077 *p* = 0.320
GSI_Diff	*r* = −0.023 *p* = 0.750	*r* = −0.047 *p* = 0.506	*r* = −0.004 *p* = 0.959	*r* = −0.027 *p* = 0.740	*r* = 0.079 *p* = 0.324	*r* = 0.131 *p* = 0.099
hsCRP_Diff	*r* = 0.030 *p* = 0.655.	*r* = 0.171* p = 0.010	*r* = 0.191* p = 0.004	*r* = −0.056 *p* = 0.485	*r* = 0.171* p = 0.022	*r* = 0.217* p = 0.004
IL6_Diff	*r* = 0.119 *p* = 0.075	*r* = 0.164* p = 0.013	*r* = 0.124 *p* = 0.063	*r* = −0.089 *p* = 0.239	*r* = 0.060 *p* = 0.427	*r* = 0.163* p = 0.030

* significant at p < 0.005,

** significant after Bonferroni correction at p < 0.004; Diff, Difference; BMI, body mass index; hsCRP, high sensitive reactive protein; IL-6, interleukin 6; BDI-II, Becks Depression Inventory; HAM-D, Hamilton Depression Score; GSI, Global severity index; Trp, Tryptophan; Kyn, Kynurenine.

## Discussion

The aim of the current analysis was to evaluate sex dependent changes in serum concentrations of Trp, Kyn and the Kyn/Trp in individuals with life-time unipolar affective disorder over a 6-week rehabilitation treatment course. At the time of admission, Trp was significantly lower and Kyn/Trp higher in women compared to men. There was a significant difference between women and men regarding the change in Trp, Kyn and Kyn/Trp over time, with increases in Kyn as well as in Kyn/Trp in men (even if controlled for relevant covariates). In both sexes, psychiatric symptoms decreased significantly over the course of treatment, reductions of HAM-D scores were significantly higher in men compared to women but did not correlate with changes in Trp breakdown. Importantly, the interaction of sex and the change in depressive symptomatology over time measured with the HAM-D significantly moderated the change in Kyn/Trp over time.

Furthermore, inflammatory marker hsCRP correlated with Kyn in both sexes, as well as with Kyn/Trp in women at the time of admission. Importantly, BMI correlated significantly with Kyn in both sexes, while it correlated with Kyn/Trp only in men. The correlations concerning the BMI were similar with the changes over time.

Sex differences in affective disorders are most consistently displayed in the increased lifetime prevalence of major depression in women compared to men. Sex differences on a neurobiological level can converge in (1) producing the same clinical outcome, (2) different outcomes or (3) result in unclear physiologic consequence [for a review see (11)]. Female and male sex hormone interactions with Kyn pathway activation have, in a small content, been described in the literature. Trp and Kyn concentrations were found approximately 15% higher in men compared to women (25). Schröcksnadel et al. (26) found Kyn/Trp rise in pregnancy, implicating that Kyn pathway activation is associated with changes in hormone levels. In addition, estrogen and progesterone have been shown to induce and androgens to inhibit Kyn pathway activity (27–30). In concordance with our results, a recent study found that women with current as well as lifetime depression had lower levels of Trp in both serum and cerebrospinal fluid compared to men (31), in the study by Elovainio et al. (32) the Kyn/Trp was found to predict depressive symptoms in women only. Furthermore, the IDO enzyme was involved in immune regulation of early atherosclerosis (33), particularly among females (34).

Only a few studies have analyzed the general effects of psychiatric treatment on the Trp-Kyn pathway, some of them evaluated the effect of antidepressant therapy with inconsistent results. Importantly, treatment response was not evaluated in all these studies as well as no stratification for sex has been performed. Reductions in Kyn pathway neurotoxic metabolites and Kyn/Trp, as well as increased Kyn/Trp after antidepressant treatment have been observed by Halaris et al. (35) and Myint et al. (15). In another study Dahl et al. (36), no changes in Kyn and metabolites in medication free depressive individuals were found after 12 weeks of treatment. Interestingly, Kyn/Trp increased during the treatment with electroconvulsive therapy (37). Sample size was small in this hitherto literature, including between 19 and 58 patients only.

Another important result of the current study was the significant positive correlation between changes in BMI and Kyn in both sexes, as well as with Kyn/Trp only in men. We could previously show a general correlation between changes in Kyn and BMI over time in a depressive extreme-group subsample of this cohort (20), this correlation could be confirmed in the current general sample. Overweight and obesity have been shown to be associated with increased immune activation and Trp breakdown in mentally healthy individuals (38–41), even more, IDO expression in adipose and hepatic tissue was higher in obese compared to lean women (42). In bipolar disorder, Kyn/Trp ratio was increased in overweight/obese compared to normal-weight patients (43). It has been assumed that overweight/obesity and metabolic syndrome, as well as associated somatic comorbidities, may be linked to the etiology, course and treatment of psychiatric disorders. The activation of the Kyn pathways in overweight/obese individuals may be an important factor of how BMI interacts with mood dysregulation.

Furthermore, also the brain-gut-axis is associated with Trp-Kyn metabolism (44). Changes in the composition of gut microbes can modulate plasma concentrations of Trp and his metabolites (45). In line with this, the microbiota is known to play a role in the regulation of serotonin synthesis, which is potentially mediated by IDO expression and stress response axis (46). Dietary and stress factors may mediate some of their effects on the gut microbiome by influencing gut permeability as well as low-level immune-inflammatory responses. Leblhuber et al. (47). Poor diet in obesity can negatively affect gut permeability, thereby contributing to an increase in gut-linked immune-inflammatory processes, with increased pro-inflammatory cytokines feeding back to further increase gut permeability.

TRP via serotonin and melatonin, is involved in the regulation of satiety and caloric intake. Intake of food rich in TRP can increase Trp availability in the body and induce the enzymatic machinery. However, this does not necessarily lead to an increase of serotonin availability in the brain because tryptophan has to pass the blood brain barrier in competition with the so-called large neutral amino acids. Nutrients rich in Trp usually also contain other amino acids in high concentrations and Trp is therefore not effectively transported into the brain. Since the release of insulin after ingestion of non-fructose carbohydrate can shift this ratio toward TRP, an individual with decreased levels of serotonin would crave carbohydrate-rich food as compensatory to serotonin depletion. In contrast, food rich in antioxidants is assumed to have two positive effects on serotonin production rates: they support serotonin biosynthesis and they slow down production of inflammatory products and associated Trp breakdown (48). After dietary intake, systemic Trp levels are regulated by hepatic trytpophan 2,3 dioxygenase (TDO). Dietary antioxidants can therefore increase brain serotonin availability but Trp does not necessarily increase in the blood. Serotonin, by regulating carbohydrate and fat intake, can lower caloric intake. Nevertheless, it is not just the amount of dietary Trp, which determines tryptophan availability, the immune system status can have a drastic influence to lower Trp levels in case of continuous activation (48). In this context it is also interesting that Kyn and the Kyn/Trp correlated positively with high levels of carbohydrate craving in a former study (49). The correlation of increased Kyn with food craving, especially carbohydrate craving, probably indicates a regulatory deficit in the maintenance of chronic inflammatory processes in obesity and BD. Sex dependent changes in stress management as well as nutritional behavior during psychiatric rehabilitation may have influenced changes in Kyn as well as Kyn/Trp in our sample.

## Limitations

As it is a naturalistic clinical study, patients were not free from medication and somatic comorbidities were present to some content. We did not analyze possible influences of psychotropic medication has due to the large inhomogeneity of drugs and doses taken. As we know from clinical studies that especially antidepressant medication might have influence on Trp and Kyn pathways, changes in Kyn as well as Kyn/Trp over time might have been associated with the respective medication. Due to the naturalistic nature and moderate sample size of our study it was not feasible to factor in all parameters that could potentially alter the association between Kyn downstream catabolits and cognitive performance. Accounting, for instance, for each and every medication would have yielded a myriad of permutations of psychopharmacological treatment combinations not fit for statistical analysis. In line with this, we also want to state that patients received a mixture of different treatments and therefore changes in the Kyn pathway cannot be linked to a specific therapeutic mechanism.

Amino acids concentrations were only measured in the serum of patients and may not accurately reflect central concentrations. However, cytokine induced increases in plasma Kyn in individuals treated with IFN- alpha correlated with increased Kyn in the cerebrospinal fluid (CSF) as well as with activated inflammatory responses and behavioral changes (50, 51). A more recent study also found significant correlations between Trp, Kyn and Kyn/Trp between serum and CSF in individuals with current as well as life-time depression (31).

The patients undergoing the psychiatric rehabilitation program differed in their stage of recovery; not all were depressive at the time of admission. However, the current study was not designed to investigate the association between a specific treatment and Kyn pathway activity, and we therefore cannot make any claims related to specific antidepressants or psychological therapies, nor on their respective effects on study outcomes.

## Conclusion

Knowledge about general changes in the Trp-Kyn pathway in individuals with affective disorders as well as about specific dysregulations in subsets of patients or differences between women and men will be of particular relevance when selecting subjects for future treatment trials targeting the Trp-Kyn pathway. The main finding of the present study was the significant difference between women and men regarding the change in Trp, Kyn and Kyn/Trp over a 6-week psychiatric treatment period, when the depression severity scores as well as general psychiatric symptoms decreased. Also, associations with BMI support previous findings of the importance of metabolic processes in affective disorders.

## Author Contributions

ER and ND: analysis of data and drafting the work; ER, KR, ND, and BR: coordination of study; DF and JG: measurement of laboratory parameters concerning Trp breakdown; All authors: revising the paper critically for important intellectual content, substantial contributions to the conception or design of the work, contributed to manuscript revision, read and approved the submitted version, interpretation of data, agree to be accountable for all aspects of the work in ensuring that questions related to the accuracy or integrity of any part of the work are appropriately investigated and resolved.

### Conflict of Interest Statement

The authors declare that the research was conducted in the absence of any commercial or financial relationships that could be construed as a potential conflict of interest.
